# Colorectal Serrated Neoplasia: An Institutional 12-Year Review Highlights the Impact of a Screening Programme

**DOI:** 10.1155/2019/1592306

**Published:** 2019-02-06

**Authors:** A. J. McCarthy, S. M. O'Reilly, J. Shanley, R. Geraghty, E. J. Ryan, G. Cullen, K. Sheahan

**Affiliations:** ^1^Department of Histopathology, St. Vincent's University Hospital, Elm Park, Dublin 4, Ireland; ^2^Centre for Colorectal Disease, St. Vincent's University Hospital, Elm Park, Dublin 4, Ireland; ^3^School of Medicine, University College Dublin, Belfield, Dublin, Ireland

## Abstract

**Background:**

As the malignant potential of sessile serrated lesions/polyps (SSL/Ps) and traditional serrated adenomas (TSAs) has been clearly demonstrated, it is important that serrated polyps are identified and correctly classified histologically.

**Aim:**

Our aim was to characterize the clinicopathological features of a series of SSL/Ps & TSAs, to assess the accuracy of the pathological diagnosis, the incidence, and the rate of dysplasia in SSL/Ps & TSAs.

**Methods:**

We identified all colorectal serrated polyps between 01/01/2004 and 31/05/2016, by searching the laboratory information system for all cases assigned a “serrated adenoma” SNOMED code. All available and suitable slides were reviewed by one pathologist, who was blinded to the original diagnosis and the site of the polyp. Subsequently discordant cases, SSL/Ps with dysplasia, and all TSAs were reviewed by a second pathologist.

**Results:**

Over a 149-month period, 759 “serrated adenoma” polyps were identified, with 664 (from 523 patients) available for review. 41.1% were reviewed by both pathologists; 15.1% (100/664) were reclassified, with the majority being changed from SSL/P to hyperplastic polyp (HYP) (66/664; 9.9%). 80.3% of these HYPs were located in the left colon, and the majority exhibited prolapse effect. There were 520 SSL/Ps (92.2%) & 40 TSAs (7.1%). The majority of SSL/Ps were in the right colon (86.7%) and were small (64.5% <1 cm), while most TSAs were in the left colon (85.7%) and were large (73.1%≥1 cm). 6.7% of SSL/Ps exhibited dysplasia, the majority of which were large (66.7%≥1 cm). Following consensus review, 13/520 (2.5%) SSL/Ps were downgraded from SSL/P with dysplasia to SSL/P without dysplasia. Detection of SSL/Ps peaked in the most recent years reviewed (87.5% reported between 2013 and 2016, inclusive), coinciding with the introduction of “BowelScreen” (the Irish FIT-based colorectal cancer screening programme).

**Conclusions:**

Awareness of, and adherence to, diagnostic criteria is essential for accurate classification of colorectal polyps.

## 1. Introduction

According to the World Health Organization (WHO), colorectal serrated lesions are a heterogeneous group of lesions characterized morphologically by a serrated architecture of the crypts, and classified histologically as hyperplastic polyps (HYPs), sessile serrated lesions/polyps (SSL/Ps) (with or without cytological dysplasia), or traditional serrated adenomas (TSAs). The features of serrated polyps of the colorectum have been discussed in comprehensive reviews, such as that by Rex *et al.* [1] and East and colleagues [2]. HYPs, SSL/Ps, and TSAs account for 83%-96%, 3-11%, and 1-7% of all serrated lesions, respectively [2–4]. Approximately 20-30% of all colorectal carcinomas have serrated polyps as their precursor lesion [5–7]. SSL/Ps progress to carcinoma via an intermediate step of SSL/P with dysplasia. These SSL/Ps with dysplasia are advanced lesions with a high risk of rapid progression to malignancy, and thus, it is vital that they are correctly identified by pathologists [8, 9].

The distinction between HYP and SSL/P can be difficult histologically, particularly in the rectum, due to the range of normal histological appearances in the rectum including some bifurcated and dilated crypts and a higher ratio of goblet cells to absorptive cells compared with other areas of the colon [10]. The architecture of rectal crypts is often distorted due to mucosal prolapse [11]. HYPs with mucosal prolapse changes (“HYPs with prolapse effect”) have been defined morphologically as hyperplastic crypts in a background of prolapsed rectal mucosa, characterized by smooth muscle proliferation in the lamina propria with entrapment and distortion of crypts [10].

Carcinomas of the serrated pathway are over-represented in studies of interval colorectal carcinomas [12], occurring due to a range of factors, including endoscopically missed precursor lesions, incompletely resected lesions, rapid progression of de novo lesions, and inadequate surveillance due to pathological misdiagnosis [13–15]. Therefore, efforts to improve pathological diagnosis of serrated polyps should help lead to a reduction in interval colorectal carcinomas [15].

With this in mind, our aim was to retrospectively review a series of serrated colorectal polyps from our institution, focusing on assessing the accuracy of pathological classification, and establishing the clinicopathological features of SSL/Ps and TSAs in our institution.

## 2. Materials and Methods

### 2.1. Case Selection

A search was performed using the laboratory information system (LIS) to identify all colorectal polyps assigned a “serrated adenoma” SNOMED code between January 1^st^ 2004 and May 31^st^ 2016. In our institution, the “serrated adenoma” SNOMED code is assigned to all SSL/Ps and TSAs (HYPs have a separate SNOMED code and were not included). Institutional ethical approval was granted.

### 2.2. Histological Review

All available and suitable haematoxylin and eosin- (H&E-) stained slides were reviewed by one pathologist (AMC), who was blinded to the original diagnosis and to the site in the colon of the polyp. All polyps were evaluated histologically and a diagnosis rendered as follows: HYP, SSL/P (with or without cytological dysplasia), TSA (with or without cytological dysplasia), or other.

#### 2.2.1. Definition of SSL/P

In the United Kingdom, the Pathology sections of the British Society of Gastroenterology (BSG) and National Health Service (NHS) Bowel Cancer Screening Programme have approved the terminology developed by Bateman and Shepherd, namely, sessile serrated lesion (SSL), with or without dysplasia [2, 16], in contrast to the WHO, which utilises the term sessile serrated adenoma/polyp (SSA/P) [17]. In our institution, we use the terminology “sessile serrated lesion/polyp” (“SSL/P”), with or without dysplasia, as agreed with our clinicians (*this terminology will be used in the remainder of this paper*). In 2012, an Expert Panel stated that “the presence of at least one unequivocal architecturally distorted, dilated, and/or horizontally branched crypt, particularly if it is associated with inverted maturation, is sufficient for a diagnosis of SSL/P” [1], and this is the definition that we used in daily practice in our institution and that we applied in our study.

#### 2.2.2. Definition of TSA

TSAs are characterized by a constellation of typical histological features, namely, striking granular eosinophilic cytoplasm, luminal serrations, the presence of ectopic crypt foci (ECF), and elongated, pencillate nuclei with evenly dispersed coarse chromatin and small inconspicuous nucleoli [18]. Some authors believe that a large proportion (the majority) of TSAs are devoid of cytological atypia (i.e., “TSA without dysplasia”), in the form of mitoses, hyperchromatic crowded nuclei displaying pleomorphism, loss of polarity, pseudostratification reaching the luminal aspects of the lining cells, or architectural features of dysplasia (crowding of glands, back-to-back arrangement, or cribriform patterns) [19].

#### 2.2.3. Definitions of Dysplasia

Histologically, SSL/P with dysplasia is identified by an abrupt transition from ordinary SSL/P to overt dysplasia. The 2010 WHO classification distinguishes two dysplasia patterns, namely, dysplasia resembling that of conventional adenomas and serrated dysplasia [17]. Recently, Liu and colleagues described in detail the morphological features of both conventional and serrated dysplasia in SSL/Ps [15].

The main characteristics of conventional adenomatous dysplasia are the predominant location of the dysplastic component on the surface (i.e., “top–down” dysplasia), with preserved non-dysplastic SSL/P at the base of the lesion and complete similarity to the dysplasia of conventional adenomas. There is no serration, and the lesional dysplastic cells are columnar with at least focal goblet cell differentiation, elongated nuclei, and pseudostratification [15].

An eosinophilic appearance at low power with tightly packed crypts is characteristic of serrated dysplasia [15]. Closely packed, small glands that occupy the full thickness of the mucosa, with occasional cribriform growth, are characteristically present. Architectural serration is less prominent, and the lesional dysplastic cells are cuboidal to low columnar with evident dysplasia, containing round vesicular nuclei, prominent nucleoli, and abundant eosinophilic cytoplasm. Mitoses are frequent, extend to the luminal surface, and can be atypical [15].

Similar to SSL/Ps, two forms of dysplasia are associated with TSAs: conventional dysplasia and serrated dysplasia (defined previously) [18, 20, 21]. Thus, we classified SSL/Ps and TSAs with dysplasia as having “conventional,” “serrated,” or “mixed conventional and serrated” dysplasia (with overlapping features of both types of dysplasia).

As per the WHO, the grade of dysplasia is reported according to a 2-tier system, either low-grade dysplasia or high-grade dysplasia. Low-grade dysplasia is an unequivocal intraepithelial neoplastic condition that must be distinguished from inflammatory or regenerative changes. It is characterized by crowded crypts arranged in parallel, without complexity, back-to-back formation, cribriforming, or budding tubules. The nuclei retain basal orientation, being confined to the bottom half of the cells. Atypical mitoses, loss of polarity, or pleomorphism is not present. The morphological criteria for high-grade dysplasia can be divided into architectural and cytological atypia, with the diagnosis being based on architecture, supplemented by an appropriate cytology. The structural features of high-grade dysplasia are characterized by complex glandular crowding and irregularity, with back-to-back glands. The lesional cells in high-grade dysplasia display loss of cell polarity or nuclear stratification and have markedly enlarged nuclei, with vesicular chromatin and prominent nucleoli. Atypical mitoses are often seen, and prominent apoptosis is frequently present.

#### 2.2.4. Definition of Discordance

Cases that had an alternative diagnosis made following this review to that made by the reporting pathologist were categorised as “discordant cases.” All discordant cases, all SSL/Ps with dysplasia, and all TSAs were reviewed by a second specialized gastrointestinal pathologist (KS), who was blinded to the original diagnosis, to the site in the colon of the polyp and to the opinion of AMC.

### 2.3. Data Collection

Pathology reports were reviewed, and various demographics were extracted (e.g., patient age and gender, site, and microscopic size of polyps). Anatomic sites were based on the original specimen requisitions submitted by endoscopists, with right-sided colonic polyps being defined as those present in the caecum, ascending colon, hepatic flexure and transverse colon, and left-sided colonic polyps being regarded as those found in, and distal to, the splenic flexure (descending colon, sigmoid colon, and rectum). Specimens that were labelled by the clinician as “random colon” and specimens without any specific designation were all categorised as “colon NOS (not otherwise specified).”

### 2.4. Data Analysis

Data was recorded and analysed using Microsoft Excel for Mac 2011 Version 14.6.4.

## 3. Results

### 3.1. Clinical Data

Over a 149-month period, 759 polyps were assigned a “serrated adenoma” SNOMED code. The H&E-stained slides of 664 of these polyps (from 523 patients) were available for review (endoscopic biopsy: 375; polypectomy: 255; endoscopic mucosal resection (EMR): 19; resection NOS: 11; piecemeal excision: 4). These polyps were from 267 male patients (51.1%) and 256 female patients (48.9%), with a median age of 64 years (range, 19-92 years) (Table 1).

### 3.2. Histological Analysis

All polyps had been reported by 9 general histopathologists; the workload of all of these pathologists comprised a large proportion of gastrointestinal biopsies.

All polyps included in this study were reviewed by one pathologist (AMC), and 41.1% of polyps (273/664) were reviewed by both pathologists (AMC & KS), with a consensus diagnosis assigned.

**Figure 1:**
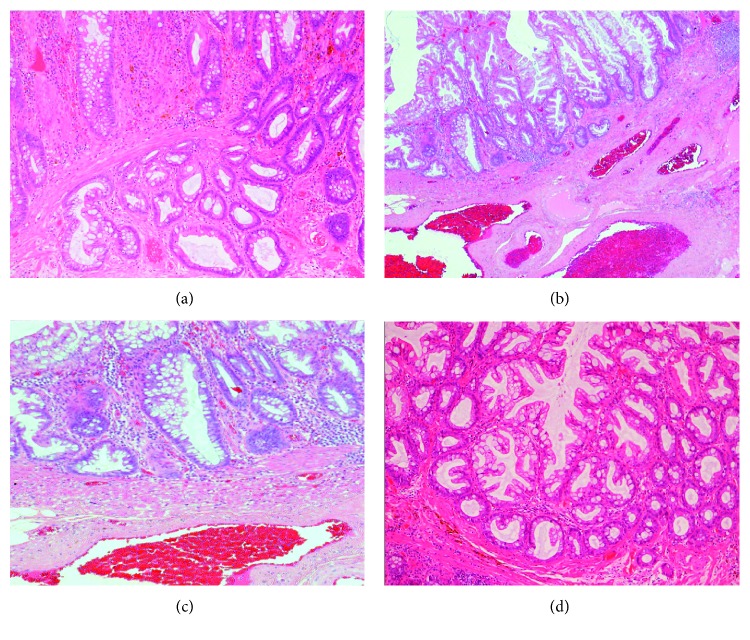
Hyperplastic polyp, with prolapse effect, characterized by dilated and congested submucosal blood vessels, thickening of the muscularis mucosae, and upward extension from the hypertrophic and splayed muscularis mucosae, with dilated crypts (a, b). Horizontal extension of crypt bases along the muscularis mucosae can be seen, mimicking the architecturally distorted, dilated, and/or horizontally branched crypts of SSL/Ps (c, d).

15.1% (100/664) of all polyps were reclassified, with the majority reclassified from SSL/P to HYP (66/664; 9.9%) (Table 2). 80.3% of these HYPs were located in the left side of the colon (53/66), and many of these exhibited prolapse effect (16/66) (Figure 1).

21 polyps (3.2%) were reclassified from TSA to conventional adenoma, and 7 polyps (1.1%) were ascribed a diagnosis of adenoma in lieu of SSL/P.

**Figure 2:**
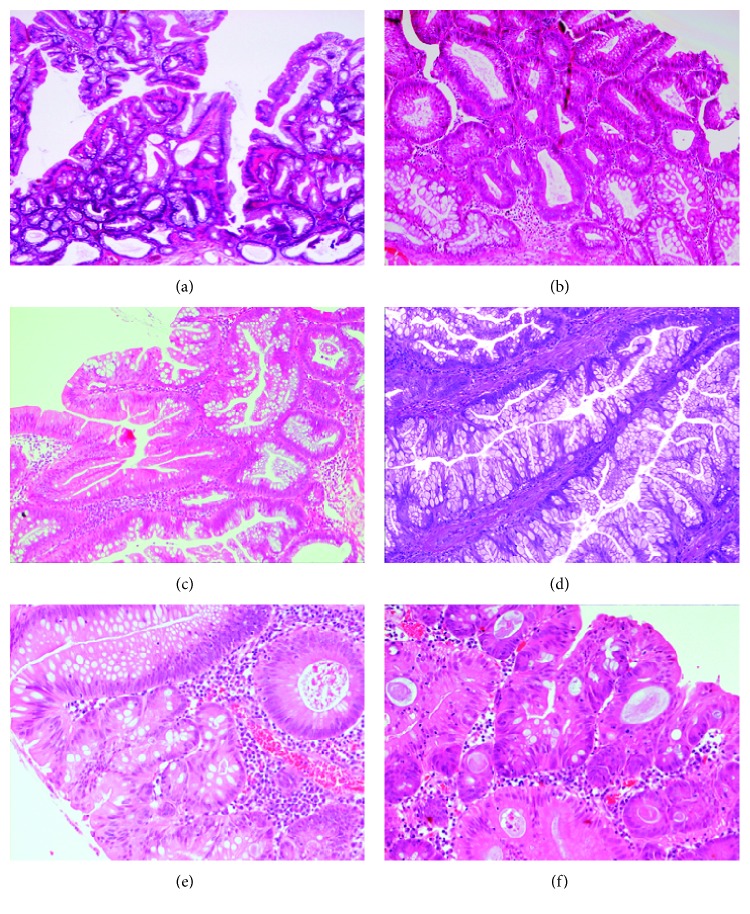
Sessile serrated lesion/polyp (SSL/P) without dysplasia (a). SSL/P with low-grade conventional adenomatous dysplasia (b). SSL/P with low-grade serrated dysplasia (c). Traditional serrated adenoma (TSA) without dysplasia (d). TSA with low-grade conventional adenomatous dysplasia (e). TSA with serrated dysplasia (f).

Following review of all 664 polyps by one or both pathologists, there were 520 SSL/Ps (78.3%, 520/664) (Figure 2(a)) & 40 TSAs (6%, 40/664) (Figure 2(d)).

86.7% of SSL/Ps were located in the right side of the colon (Table 3), with the majority being found in the ascending colon (200/520; 38.5%). 64.5% were small in size (<1 cm), with a mean size of 8.1 mm (median, 8.1 mm; range, 1–32 mm).

Following consensus review, 13/520 (2.5%) SSL/Ps were downgraded from SSL/P with dysplasia to SSL/P without dysplasia. Overall, 6.7% of SSL/Ps exhibited dysplasia (35/520), all demonstrating low-grade dysplasia. The majority of these were found in the right side of the colon (28/35; 80%), with most being located in the transverse colon (9/35; 25.7%). SSL/Ps with dysplasia (66.7%) were mainly large in size (≥1 cm), with a mean size of 11.3 mm (range, 3–30 mm) (Table 3).

The majority of SSL/Ps exhibited conventional adenomatous dysplasia (25/35; 71.4%) (Figure 2(b), representative image), 3 cases (8.6%) demonstrated serrated dysplasia (Figure 2(c), representative image), and 20% (7/35) displayed a mixture of both conventional and serrated dysplasia.

**Figure 3:**
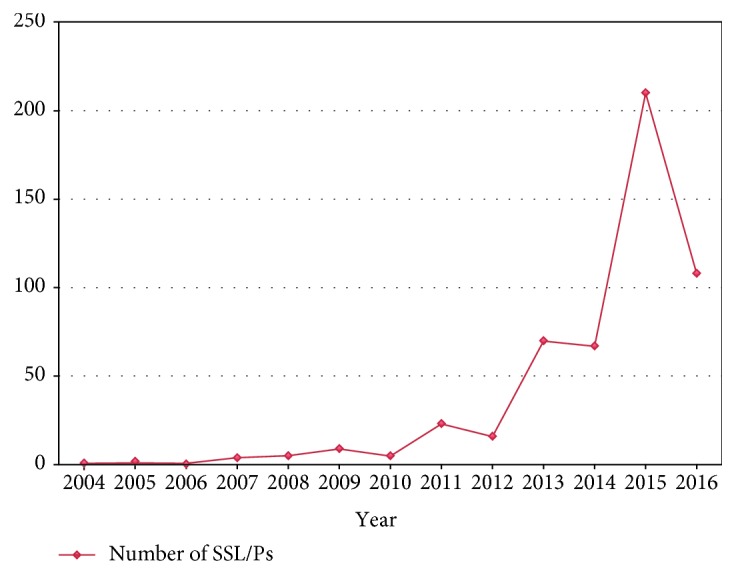
The number of SSL/Ps detected between January 1^st^ 2004 and May 31^st^ 2016, with detection of SSL/Ps peaking in the most recent years included in this review (87.5% reported between 2013 & 2016, inclusive). This coincided with the introduction of “BowelScreen” (the Irish colorectal cancer screening programme).

Detection of SSL/Ps peaked in the most recent years reviewed (87.5% reported between 2013 and 2016, inclusive), coinciding with the introduction of “BowelScreen” (the Irish colorectal cancer screening programme, http://www.bowelscreen.ie) (Figure 3).

85.7% of TSAs were located in the left side of the colon, with the majority being found in the rectum or sigmoid colon (32/40; 80%). 73.1% were large in size (≥1 cm), with a mean size of 18.6 mm (range, 2–60 mm) (Table 4). 67.5% of TSAs exhibited dysplasia (27/40), with low-grade dysplasia in 60% (24/27 TSAs with dysplasia) and high-grade dysplasia in 7.5% (3/27 TSAs with dysplasia) of all TSAs. The majority of TSAs exhibited conventional adenomatous dysplasia (26/27; 96.3%) (Figure 2(e), representative image), and 1 case (3.7%) demonstrated serrated dysplasia (Figure 2(f), representative image).

Table 5 highlights the key features of SSL/Ps and TSAs.

## 4. Discussion

We reviewed a large series of serrated polyps over a 12-year period, focusing on the histological diagnosis of SSL/Ps and TSAs, with and without dysplasia. Detection of SSL/Ps peaked in the most recent years included in this review (87.5% reported between 2013 & 2016, inclusive). This coincided with the introduction of “BowelScreen” (the FIT-based Irish colorectal cancer screening programme) and improved recognition of this entity by histopathologists. Similarly, Chetty and colleagues documented increasing awareness in their institution of SSL/Ps as an entity over a 4-year period [22]. With the continued rollout of “BowelScreen” across Ireland, and similar colorectal cancer screening programmes in many other countries, in conjunction with improved colonoscopy techniques, pathologists who report specimens resulting from screening programmes can anticipate encountering SSL/Ps on a regular basis.

Furthermore, this study clearly shows how challenging it can be to distinguish SSL/Ps from HYPs, as there are often only subtle differences, with 66/664 polyps in our cohort (9.9%) being reclassified from SSL/P without dysplasia to HYP following consensus review. In a related study, Gill and colleagues reviewed a large series of right-sided lesions originally diagnosed as HYPs and recategorised 30–64% of HYPs over a 4-year period to SSL/Ps, emphasising again how difficult the distinction between HYP and SSL/P can be [23]. Reviewing all right-sided serrated/hyperplastic polyps was not included in our study design, and thus, we have no information on the rate of under-diagnosis of SSL/Ps in our institution.

Identification of architecturally distorted, dilated, and/or horizontally branched crypts (“L,” “boot,” or “anchor”-shaped crypts), in association with excessive/hyper-serration in the basal half of crypts, is required for a diagnosis of SSL/P (Table 6). However, there are differences of opinion between pathologists in the United States, the United Kingdom, and other parts of Europe regarding the pathological features required to make a diagnosis of an SSL/P. An expert panel, including gastroenterologists, scientists, and pathologists, recommended that serrated polyps with as few as one of these SSL/P-type crypts should be diagnosed as an SSL/P [1]. However, the WHO states that a diagnosis of SSL/P should be made when a serrated polyp shows 2 or 3 contiguous SSL/P-type crypts [24]. Kolb *et al.* found that using the expert panel criteria resulted in improved interobserver agreement and in an approximately 7% increase in the diagnosis of SSL/P when compared with the WHO criteria [25]. Bettington and colleagues analysed 6340 polyps and reported an SSL/P incidence of 12.1% when WHO criteria were applied, versus 14.7% when using the expert panel criteria. They reported that serrated polyps with any SSL/P-like crypts (expert panel criterion) had clinical features more like SSL/Ps than HYPs (more proximal location, larger size, etc.), and they concluded that only 1 abnormal crypt is necessary for the diagnosis of SSL/P, independent of size and location [26]. In our institution, we apply the recommendations of the expert panel consensus document.

In contrast to SSL/Ps, HYPs are characterized by simple, elongated crypts with a serrated structure in the upper half of the crypts, with some proliferation in the basal (non-serrated) part of the crypts (Table 6). Particularly challenging is distinguishing SSL/Ps from HYPs with prolapse effect, and this diagnostic conundrum has been previously documented in the literature [10, 11, 27, 28]. Prolapse complicating HYPs can result in horizontal extension of crypt bases along the muscularis mucosae, mimicking the architecturally distorted, dilated, and/or horizontally branched crypts of SSL/Ps. Histologically, prolapse is characterized by thickening of the muscularis mucosae, with upward extension from the hypertrophic and splayed muscularis mucosae and fibromuscular obliteration of the lamina propria, with dilated crypts [11]. Awareness of this pitfall, particularly in the rectum, will enable histopathologists to render the correct diagnosis.

It is known that SSL/Ps with dysplasia are precursors for interval colorectal carcinomas, and that these lesions are rapidly progressive, difficult to detect endoscopically, commonly incompletely resected, and occasionally misdiagnosed histologically [15, 29]. Due to this significant risk, it is thought that some SSL/Ps should be clinically managed in the same fashion as conventional adenomas, with the British Society of Gastroenterology recommending that patients with certain SSL/Ps (those ≥10 mm or serrated lesions harbouring dysplasia, including TSAs) should be offered a one-off colonoscopic surveillance examination at 3 years [2]. However, such a strategy relies on the ability of histopathologists to reproducibly distinguish SSL/Ps from other serrated polyps, especially those without dysplasia, namely, HYPs, to correctly triage patients. In our study, agreement between the reviewing pathologist's/pathologists' and the original pathologists' histological diagnosis was reached for 85% (564/664) of all polyps reviewed. 100/664 (15.1%) serrated polyps originally classified as SSL/Ps were reclassified as HYPs, adenomas, or benign polyps NOS following consensus review. Strict adherence to the morphological features required [16, 30] for a diagnosis of SSL/P should help to reduce interobserver variability between pathologists.

In a review of SSL/Ps from 2139 patients, Lash *et al.* identified low-grade dysplasia and high-grade dysplasia in 12% and 2.1% of their patients with SSL/Ps, respectively [3]. Yang and colleagues reported 13,072 SSL/Ps, the majority of which (95%) were negative for dysplasia [9]. 4.6% of their SSL/Ps had low-grade cytological dysplasia, and 0.35% had high-grade cytological dysplasia. Similar to Yang *et al.*, a low rate of SSL/Ps with dysplasia (6.7%) is confirmed in our institution, with conventional adenomatous dysplasia, serrated dysplasia, and a mixture of both conventional and serrated dysplasia in 71.4%, 8.6%, and 20% of SSL/Ps with dysplasia, respectively.

The concept of dysplasia in TSAs is controversial. Many pathologists consider TSAs to be inherently dysplastic and routinely report low-grade dysplasia in TSAs mainly based on elongated, pencillate nuclei [19]. Bettington and Chetty, among others, propose that, although the ordinary TSA is undoubtedly neoplastic, it does not have inherent cytological dysplasia, as the eosinophilic cells of an ordinary TSA are not overtly atypical, do not have mitoses, have minimal proliferative activity by Ki-67 staining, and do not show other immunohistochemical changes to suggest dysplasia (i.e., no abnormal staining with *β*-catenin, p53, and/or p16) [21, 31, 32]. Thus, the major issue for the practicing pathologist is to recognize areas of overt (e.g. adenomatous) dysplasia arising in a TSA and to bring this to the attention of the endoscopist [19]. With this in mind, following consensus review of our TSAs, we report low-grade dysplasia in 60%, and high-grade dysplasia in 7.5% of TSAs included in our series.

There are some limitations to our study. We did not retrieve and review all polyps that were classified as HYPs, to assess how many would be amended to SSL/P on review. We chose not to interrogate this area, as this has been previously studied and published by other authors [33–41]. Instead, we approached this topic from the opposite viewpoint, by reviewing a large series of serrated polyps already classified as SSL/Ps or TSAs, and focusing on the accuracy of the histological diagnoses of SSL/P and TSA in our institution. We were also keen to establish our rate of dysplasia in SSL/Ps and TSAs, to compare it with that quoted in published literature. Although accompanying ancillary molecular testing would likely be illuminating, it was not employed as this is a purely morphological study highlighting the necessity to strictly adherence to robust diagnostic criteria.

## 5. Conclusion

As the malignant potential of SSL/Ps and TSAs has been clearly established, it is important that serrated polyps are identified and correctly classified histologically. It is therefore essential for all pathologists to strictly adhere to diagnostic criteria and to be aware of pitfalls in diagnosis. In particular, as has been established in the literature, failure to identify serrated polyps with dysplasia may result in inadequate surveillance and thus increases the risk of interval colorectal carcinoma.

## Figures and Tables

**Table 1:** Demographics of patients included in this study (664 polyps from 523 patients), all of whom had at least one polyp originally reported as either SSL/P or TSA.

Gender *n* (%)	Male	267 (51.1%)
	Female	256 (48.9%)

Age (years)	Median	64
	Range	19–92

Abbreviations: SSL/P: sessile serrated lesion/polyp; TSA: traditional serrated adenoma.

**Table 2:** Reclassification of serrated polyps (SSL/Ps and TSAs) following consensus review by two pathologists; (out of 664 reviewed).

Change in classification	Number (%)
Changed from SSL/P to HYP	66 (9.9%)
Changed from SSL/P to adenoma	7 (1.1%)
Changed from SSL/P to benign polyp NOS	1 (0.2%)
Changed from mixed serrated polyp to adenoma	5 (0.8%)
Changed from TSA to adenoma	21 (3.2%)

Abbreviations: SSL/P(s): sessile serrated lesion(s)/polyp(s); TSA(s): traditional serrated adenoma(s); HYP: hyperplastic polyp; NOS: not otherwise specified.

**Table 3:** Characteristics of SSL/Ps identified over a 12-year period at our institution; *n* = 520.

Parameter	All SSL/Ps	SSL/Ps without dysplasia	SSL/Ps with dysplasia (all LGD)
Number (*n*, %)	520	485 (93.3%)	35 (6.7%)
Age of patient (years)			
Median	63	63	69
Range	19-84	19-84	47-83
Site (*n*, %)			
Right colon	451 (86.7%)	423 (87.2%)	28 (80%)
Left colon	62 (11.9%)	55 (11.3%)	7 (20%)
Colon NOS	7 (1.4%)	7 (1.4%)	—
Size (mm)			
Median	8	8	10
Range	1-32	1-32	3-30
Size category (*n*, %)			
< 1 cm	167 (64.5%)	162 (66.4%)	5 (33.3%)
≥1 cm	92 (35.5%)	82 (33.6%)	10 (66.7%)

Abbreviations: SSL/Ps: sessile serrated lesions/polyps; NOS: not otherwise specified; LGD: low-grade dysplasia.

**Table 4:** Characteristics of TSAs identified over a 12-year period at our institution; *n* = 40.

Parameter	All TSAs	TSAs without dysplasia	TSAs with dysplasia
Number (*n*, %)	40	13 (32.5%)	27 (67.5%)
Age of patient (years)			
Median	67	61	68
Range	34-92	34-86	44-92
Site (*n*, %)			
Right colon	4 (10%)	2 (15.4%)	2 (7.4%)
Left colon	34 (85%)	9 (69.2%)	25 (92.6%)
Colon NOS	2 (5%)	2 (15.4%)	—
Size (mm)			
Median	13	10	23.2
Range	2-60	2-20	4-60
Size category (*n*, %)			
<1 cm	7 (26.9%)	4 (44.4%)	3 (17.6%)
≥1 cm	19 (73.1%)	5 (55.6%)	14 (82.4%)
Dysplasia grade (*n*, %)			
Low	24 (60%)	N/A	24 (88.9%)
High	3 (7.5%)	N/A	3 (11.1%)
Type of dysplasia (*n*, %)			
Conventional	26 (65%)	N/A	26 (96.3%)
Serrated	1 (2.5%)	N/A	1 (3.7%)

Abbreviations: TSAs: traditional serrated adenomas; NOS: not otherwise specified; N/A: not applicable.

**Table 5:** Characteristics of SSL/Ps & TSAs identified over a 12-year period at our institution.

Parameter	All SSL/Ps	All TSAs
Number (*n*, %)	520	40
Age of patient (years)		
Median	63	67
Range	19-84	34-92
Site (*n*, %)		
Right colon	451 (86.7%)	4 (10%)
Left colon	62 (11.9%)	34 (85%)
Colon NOS	7 (1.4%)	2 (5%)
Size (mm)		
Median	8	13
Range	1-32	2-60
Size category (*n*, %)		
<1 cm	167 (64.5%)	7 (26.9%)
≥1 cm	92 (35.5%)	19 (73.1%)
Dysplasia (*n*, %)		
Low	35 (6.7%)	24 (60%)
High	—	3 (7.5%)
Type of dysplasia (*n*, %)		
Conventional	25 (71.4%)	26 (65%)
Serrated	3 (8.6%)	1 (2.5%)
Mixed	7 (20%)	—

Abbreviations: SSL/Ps: sessile serrated lesions/polyps; TSAs: traditional serrated adenomas; NOS: not otherwise specified.

**Table 6:** Histological features of hyperplastic polyps with prolapse, sessile serrated lesions/polyps, and traditional serrated adenomas [1, 11, 18].

HYP with prolapse effect	HYP: (i) Simple, elongated crypts (ii) Serrated structure in the upper half of the crypts (iii) Some proliferation in the basal (non-serrated) part of the crypts Prolapse: (i) Thickening of the muscularis mucosae (ii) Upward extension from the hypertrophic & splayed muscularis mucosae (iii) Fibromuscular obliteration of the lamina propria, with dilated crypts

SSL/P	(i) At least one unequivocal architecturally distorted, dilated, &/or horizontally branched crypt (“L,” “boot,” or “anchor”-shaped crypt) (ii) Inverted maturation (excessive/hyper-serration in the basal half of crypts)

TSA	(i) Striking granular eosinophilic cytoplasm (ii) Luminal serrations (iii) Ectopic crypt foci (iv) Elongated, pencillate nuclei with evenly dispersed coarse chromatin & small inconspicuous nucleoli

*Abbreviations:* HYP: hyperplastic polyp; SSL/P: sessile serrated lesion/polyp; TSA: traditional serrated adenoma.

## Data Availability

All of the data used to support the findings of this study are included within the article.
